# Jeopardy to life and limb—using immunogenotyping to characterize inflammatory phenotypes: a case report

**DOI:** 10.1093/rap/rkaf020

**Published:** 2025-02-22

**Authors:** Kirishananth Rajaseelan, Saad Ahmed, Frances Hall, Emese Balogh, Dinakantha Kumararatne, Anita Chandra, Ania Manson

**Affiliations:** Department of Medicine, Addenbrooke’s Hospital, Cambridge, UK; Department of Medicine, Addenbrooke’s Hospital, Cambridge, UK; Department of Medicine, Addenbrooke’s Hospital, Cambridge, UK; Department of Medicine, Addenbrooke’s Hospital, Cambridge, UK; Department of Medicine, Addenbrooke’s Hospital, Cambridge, UK; Department of Medicine, Addenbrooke’s Hospital, Cambridge, UK; Department of Clinical Immunology, Cambridge University Hospitals NHS Foundation Trust, Cambridge, UK; Department of Medicine, University of Cambridge, Cambridge, UK; Department of Medicine, Addenbrooke’s Hospital, Cambridge, UK

Key message

- Mechanistic thinking is paramount when a clinical phenotype does not map to a recognized disorder.

Dear Editor, NFκB is an inducible transcription factor with a critical role in immune cell survival and differentiation. The NFκB pathway consists of the canonical and non-canonical pathways, the former regulating proinflammatory genes and the latter involving TNF receptor superfamily members and contributing to immune cell development and homeostasis, amongst other functions. We describe the case of a 59-year-old male with progressive postoperative necrotizing fasciitis in whom genomic analysis revealed a heterozygous truncating mutation in the *NFKB1* gene. The uniqueness of our case includes its rarity but also the significant collateral damage that can occur when inflammatory cascades escape homeostatic control due to defects in the pathway, which regulates un-opposed inflammation.

A 59-year-old male underwent surgery for Dupuytren’s contracture of the left hand. Four days later, he complained of intense pain, attributed to the pressure dressing. Seven days after surgery, a purulent exudate developed. Progressive tissue damage occurred in the hand and forearm (Fig. 1), with crops of dusky, red lesions appearing at the wound margins. The patient had a history of psoriasis. His mother had rheumatoid arthritis. His brother had died in his mid-forties from sepsis, having developed necrotizing fasciitis in the right inguinal region and bilateral antecubital fossae. This had followed appendicectomy. There had been no response to courses of antibiotics but transient improvement with a course of prednisolone. Further relevant history is outlined in Supplementary Data S1, available at *Rheumatology* online.

**Figure 1.**
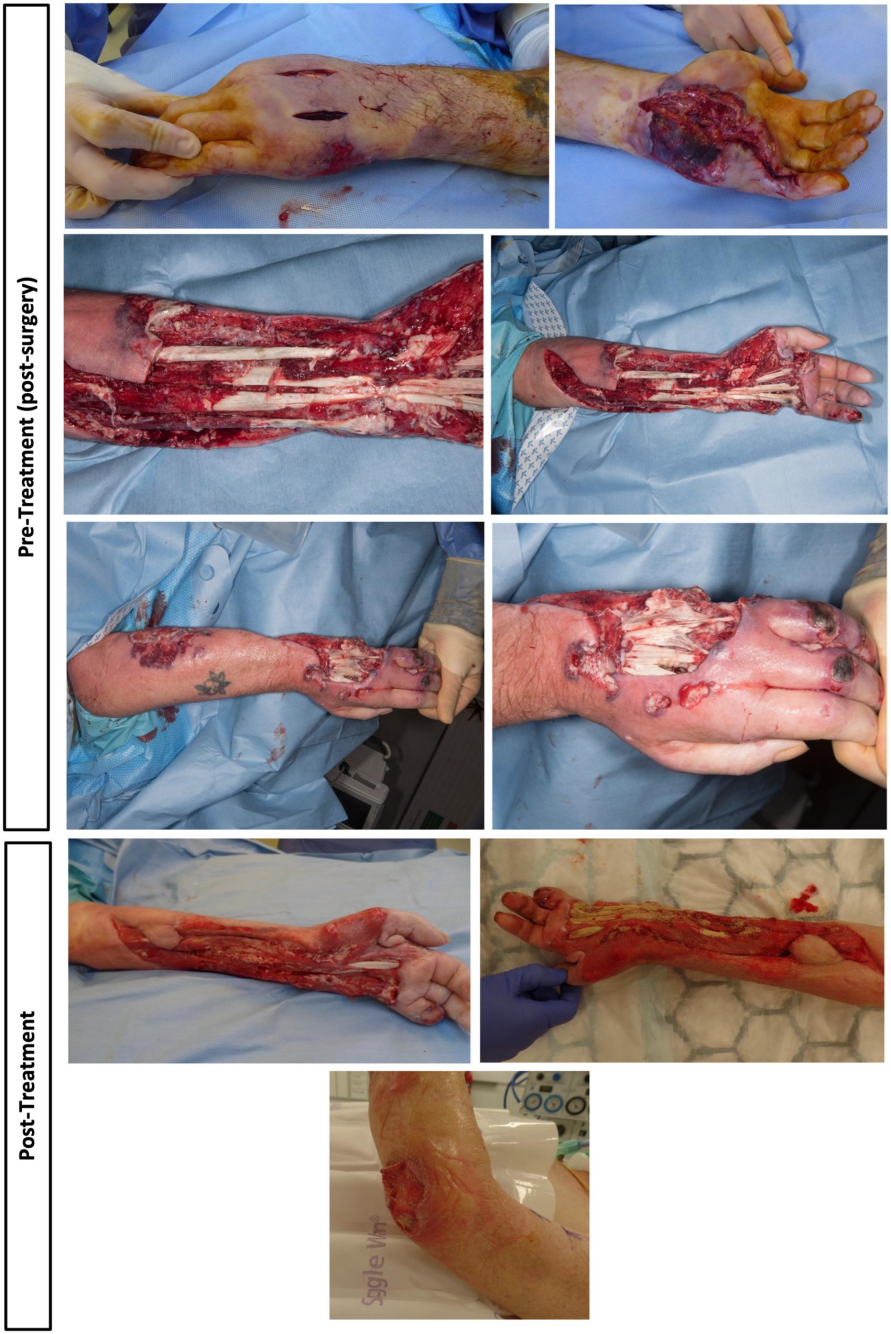
Pre- and post-treatment (prednisolone, IVIG and Anakinra)

The patient was febrile at 38.1°C and had transient trace proteinuria with an estimated glomerular filtration rate dropping to a nadir of 74 ml/min. There was no other tissue involvement other than right wrist synovitis, which the patient developed.

Investigations revealed an acute phase response with transaminitis. The patient’s haemoglobin dropped to 87 g/l with a neutrophilia at 23.81 × 10^9^/l. Low IgM levels were identified (0.2 g/l). Antiphospholipid antibody tests returned transiently positive anti-cardiolipin IgG (11.1 IU/ml) and Lupus Anticoagulant (1.28 elevated ratio) tests only. Wound cultures were unrevealing. Autoimmune serology and other blood test results are presented in Supplementary Table S1, available at *Rheumatology* online.

A biopsy reported necrotizing ulceration with oedema and neutrophilic infiltration, involving the dermis, subcutis and skeletal muscle, suggesting potential pyoderma gangrenosum (PG). Stains for fungi and mycobacteria were negative.

This case was discussed at the Eastern Network for Rare Autoimmune Disease (ENRAD) meeting. Differential diagnosis included autoinflammatory disease, primary IgM deficiency with invasive infection, small-vessel vasculitis and antiphospholipid syndrome (APS). A primary autoinflammatory condition was the most probable, given the aberrant, ongoing activity of the inflammatory cascade, following surgical tissue injury. There was no obvious infectious driver. There were no serological or histological correlates of small-vessel vasculitis or connective tissue disease, except weak (and transient) anti-phospholipid antibody signals. This patient and his brother both appeared to have had an unremarkable medical history until middle age, but then developed a destructive acute inflammatory response triggered by tissue injury. The consensus strategy from ENRAD was to suppress acute inflammatory activity and to taper corticosteroid, with the anticipation that immunosuppression could be withdrawn after complete wound healing. Use of anakinra 100 mg s/c was supported.

After the addition of anakinra to prednisolone (50 mg) and ciclosporin (125 mg BD), progression of pyoderma was rapidly arrested with high-dose corticosteroids. A series of (>20) debridements were performed with ongoing steroid treatment. A course of immunomodulatory IVIG (2 g/kg over 5 days every month), followed by ciclosporin (due to concerns over reinfection risk) and anakinra, enabled reduction of prednisolone to optimize wound healing prior to amputation. This therapeutic strategy arrested local inflammation and enabled tissue healing (Fig. 1). The efficacy of IVIG and anakinra enabled salvage of the upper arm; a below-elbow amputation was performed. Histology showed mild perivascular chronic inflammation.

Anakinra was continued for 8 months, during which time the below-elbow stump healed well, and prednisolone was stopped, followed by Anakinra. There was no disease recurrence.

The genomic test results for an Immunodeficiency and Autoimmune panel revealed an NKFB1 gene mutation (c.1190dupG; p.(T398HfsTer9)). This heterozygous sequence change is caused by a duplication, resulting in a frameshift mutation at codon encoding Threonine at position 398; the new reading frame ends in a premature STOP codon 9 positions downstream. The mutation was confirmed using the GEMINI PID Panel Next Generation Sequencing (NGS) of the coding region of a set of genes associated with primary immune deficiency using the Illumina TruSight One sequencing panel. Mutations causing similar frameshifts in the NFκΒ1 genes are pathogenic on the Human Gene Mutation Database [1].

This case highlights the importance of mechanistic thinking in situations where the clinical features do not map to a recognized disorder. Inflammation at a surgical site is usually caused by local infection. However, in this case, infection was unlikely due to failure to identify a plausible pathogen and due to the absence of response to several antibiotics. The patient had a selective deficiency of IgM. Low IgM is relatively common, often with no clinical significance, though associated with recurrent respiratory infections and some autoimmune diseases, but not necrotizing fasciitis. PG is a reactive neutrophilic dermatosis, which involves upregulation of chemotactic factors, increased matrix metalloproteinase expression and T-cell clonal expansion [2]. Diagnostic delay is common, and 50% of cases are secondary to a systemic condition [3]; treatment is guided by severity and extent with a limited evidence base.

The patient had a history of psoriasis, associated with an aberrant Th17 response and neutrophilic infiltration in lesions [4]. A range of autoinflammatory conditions with associated skin necrosis have been reported, but this case lacked other clinical features. This case suggested an inherited defect in the acute inflammatory response, which was triggered following tissue damage.

In this case, it appears that an inflammatory response was triggered, then disproportionately amplified, following surgery. In the patient’s brother, surgery for appendicitis was the trigger; it is unknown whether appendicitis was confirmed histologically.

NFkB mutations have been associated with diverse phenotypes [5]. Post-surgical hyperinflammatory responses with enhanced inflammasome activation is predisposed by pArg157X stop-gain mutation [5]. *NFKB1* mutations have also been associated with common variable immunodeficiency [6].

In summary, organ-threatening inflammation transpired to be a manifestation of autoinflammation in this patient, and probably in his brother. Sequence changes in the *NFkB1* gene were discovered and are plausible links with the apparent triggering—and perpetuation—of inflammation, in response to injury. Functional studies of these variants are warranted to further explore causality. This case provides an example of utilizing immunogenotyping to generate pathogenic hypotheses to explain unusual inflammatory phenotypes.

## Supplementary Material

rkaf020_Supplementary_Data

## Data Availability

The data that support the findings of this study are available on request from the corresponding author.
