# Supplementary material for: Jeopardy to life and limb—using immunogenotyping to characterize inflammatory phenotypes: a case report
Source: Rheumatol Adv Pract. 2025 Feb 22;9(1):rkaf020. doi: 10.1093/rap/rkaf020 (PMC11879314; doi:10.1093/rap/rkaf020)
Supplement: rkaf020_Supplementary_Data [file rkaf020_supplementary_data.zip › 24-276 Supplementary Material.docx]

**Supplementary Data S1. Further history**

An inflammatory response is typically triggered via pattern recognition receptors (PRR) on sentinel cells which detect either evidence of microbial infection (pathogen-associated molecular patterns; PAMPs) or tissue damage (danger-associated molecular patterns; DAMPs). This triggering leads to activation of the inflammasome, followed production of interleukin-1, a local vascular response an acute-phase response, including fever. The delivery of mediators into the affected tissue is primarily designed to contain and destroy pathogens. However, there is significant collateral damage. For this reason, inflammatory cascades are designed to be time-limited or actively inhibited in the absence of ongoing stimulation of PRRs

A range of autoimmune syndromes can cause rapid tissue necrosis including small vessel vasculitis, systemic lupus erythematosus, dermatomyositis. However, clinical, and serological features of these vasculitides were absent.

Major clinical features include recurrent infections, especially of the respiratory tract (which occurs in 95% of patients with CVID) or gut (in approximately 50% of patients), and autoimmune disease, which occurs in over a quarter of patients diagnosed with CVID. Approximately 10% have lymphoproliferative disease.

Functional antibody assessment revealed poor pneumococcal response, antipneumococcal antibodies (normal against 8/13 pneumococcal serotypes greater than, or equal to 0.35 μg/ml), adequate IgG specific for tetanus toxoid (range from 0.140-0.660 IU/ml) and detectable IgG specific for measles, mumps, rubella, Haemophilus influenza b (suboptimal, 0.67 µg/ml) and SARS-CoV2. A functional cytokine assay panel, with a range of TLR stimuli or polyclonal T cell activator, anti-CD3 antibody revealed excessive production of interleukin 6 and interleukin 1.

**Supplementary Table S1: Blood Investigation Results and Autoimmune Serology**

| **Blood Results** | | |
| --- | --- | --- |
| Haemoglobin | 87 | g/L |
| Platelet Count | 184 | x10^9^/L |
| Neutrophil Count | 23.81 | x10^9^/L |
| C-Reactive Protein | 301 | mg/L |
| Erythrocyte Sedimentation Rate | 109 | mm/h |
| Alkaline Phosphatase | 180 | U/L |
| Albumin | 15 | g/L |
| Alanine Transaminase | 97 | U/L |
| eGFR | >=90 | ml/min/1.73m^2^ |
| **Autoimmune Serology** | | |
| ANA | Negative (ELISA and Hep2) |  |
| RhF | 13 | iu/ml |
| Anti-proteinase 3 | Negative | u/ml |
| Anti-myeloperoxidase | Negative | iu/ml |
| IgG | 10.08 | g/L |
| IgA | 1.22 | g/L |
| IgM | 0.20 | g/L |
| Serum Electrophoresis | Normal | g/L |
| Serum Free Light Chains | Normal | mg/L |
